# The Prawn *Macrobrachium vollenhovenii* in the Senegal River Basin: Towards Sustainable Restocking of All-Male Populations for Biological Control of Schistosomiasis

**DOI:** 10.1371/journal.pntd.0003060

**Published:** 2014-08-28

**Authors:** Amit Savaya Alkalay, Ohad Rosen, Susanne H. Sokolow, Yacinthe P. W. Faye, Djibril S. Faye, Eliahu D. Aflalo, Nicolas Jouanard, Dina Zilberg, Elizabeth Huttinger, Amir Sagi

**Affiliations:** 1 Department of Life Sciences and the National Institute for Biotechnology in the Negev, Ben-Gurion University, Beer Sheva, Israel; 2 French Associates Institute for Agriculture and Biotechnology of Drylands, Jacob Blaustein Institute for Desert Research, Ben-Gurion University, Sede-Boqer, Israel; 3 Department of Biology, Hopkins Marine Station, Stanford University, Palo Alto, California, United States of America; 4 Université Gaston Berger, Saint-Louis, Senegal; 5 University Cheikh Anta Diop, Fann, Dakar, Senegal; 6 Centre de Recherche Biomédicale Espoir Pour La Santé, Sor, Saint-Louis, Senegal; 7 The 20|20 Initiative, Pasadena, California, United States of America; George Washington University School of Medicine and Health Sciences, United States of America

## Abstract

Early malacological literature suggests that the outbreak of schistosomiasis, a parasitic disease transmitted by aquatic snails, in the Senegal River basin occurred due to ecological changes resulting from the construction of the Diama dam. The common treatment, the drug praziquantel, does not protect from the high risk of re-infection due to human contact with infested water on a daily basis. The construction of the dam interfered with the life cycle of the prawn *Macrobrachium vollenhovenii* by blocking its access to breeding grounds in the estuary. These prawns were demonstrated to be potential biological control agents, being effective predators of *Schistosoma*-susceptible snails. Here, we propose a responsible restocking strategy using all-male prawn populations which could provide sustainable disease control. Male prawns reach a larger size and have a lower tendency to migrate than females. We, therefore, expect that periodic restocking of all-male juveniles will decrease the prevalence of schistosomiasis and increase villagers' welfare. In this interdisciplinary study, we examined current prawn abundance along the river basin, complemented with a retrospective questionnaire completed by local fishermen. We revealed the current absence of prawns upriver and thus demonstrated the need for restocking. Since male prawns are suggested to be preferable for bio-control, we laid the molecular foundation for production of all-male *M. vollenhovenii* through a complete sequencing of the insulin-like androgenic gland-encoding gene (IAG), which is responsible for sexual differentiation in crustaceans. We also conducted bioinformatics and immunohistochemistry analyses to demonstrate the similarity of this sequence to the IAG of another *Macrobrachium* species in which neo-females are produced and their progeny are 100% males. At least 100 million people at risk of schistosomiasis are residents of areas that experienced water management manipulations. Our suggested non-breeding sustainable model of control—if proven successful—could prevent re-infections and thus prove useful throughout the world.

## Author Summary

Schistosomiasis is a chronic parasitic disease that infects millions of people, especially in Africa. Schistosomes are transmitted by direct contact with water sources infested by freshwater snails, which are intermediate hosts for the parasite. The cure in humans is a drug, praziquantel, that kills the mature parasites inside the human body. The main problem with controlling the parasite by drug treatment is the high re-infection rate, since individuals are in contact with infected water on a daily basis. To efficiently combat the disease, an integrated management program is needed that includes control of infection in the intermediate host snails. We suggest the use of non-migrating, all-male populations of freshwater prawns that efficiently prey on these snails. Here, we describe the case of the Senegal River basin as an example of human actions (dam construction) that resulted in severe ecosystem changes, including exclusion of the native river prawns and expansion of snails hosting schistosomiasis. We have conducted an interdisciplinary study that documents the reduction of prawn abundance in the Senegal River and lays the molecular foundation for technology to produce all-male prawn populations to be used as part of an integrated disease control program, including both periodic stocking of juvenile prawns and chemotherapy.

## Introduction

Schistosomiasis is a chronic parasitic disease caused by blood flukes of the genus *Schistosoma*, which are dependent on two hosts to complete their life cycle, an intermediate host (a freshwater snail) and a definitive host (a vertebrate). The adult parasites can live for decades and cause increasing damage to organ tissues (bladder, liver or intestine) and can result in mortality of the host [1]. One of the most heavily infected areas in the world is the Senegal River basin in which the outbreak of the disease was reported following the construction of the Diama dam, ∼50 km from the mouth of the river, in 1986. The dam is a saltwater barrier and was built to support agricultural expansion in the delta and upriver by preventing saltwater intrusion during the dry season [2]. As a result of dam construction, the Senegal River basin ecosystem experienced major changes, such as habitat expansion for fresh water species, like aquatic snails hosting schistosomiasis [3]–[6]. Since the appearance of the dam, rates of *Schistosoma haematobium* infection have risen from 0–3.6% to 11.5%, and from 10.4–27.2% to 51.6% in different areas of the river basin [7]. Moreover, while *S. mansoni* was absent in the river basin before the construction of the dam, it was first reported 18 months after the dam was completed, with the associated infection rates now reaching up to 71.8% in some villages [7]. The ecological changes related to the separation of the upriver region from the estuary also are unfavorable for catadromous species, such as the native river prawn *Macrobrachium vollenhovenii*.

*M. vollenhovenii* is a decapod crustacean belonging to the Palaemonidae family, endemic to the west coast of Africa from the Senegal River in the north to Angola in the south [8]–[10]. The northern habitat border of the prawn, the Senegal River basin, supported artisanal prawn fishery extending from the coast to more than 400 km inland prior to dam construction [11]. This natural habitat was confronted with an insurmountable challenge following construction of the dam due to the prawn's dependence on brackish water and access to the estuary to complete their life cycle. Ovigerous females of this species must migrate to the estuary in order to release their larvae, which in turn complete their larval development period in brackish water before migrating upriver as post-larvae [12]–[14]. The increased snail numbers after construction of the dam could be explained by a slowing of the river flow and decreased saltwater intrusion, thereby expanding regions of suitable habitat for the snails. This, together with the human migration seeking employment in the expanded rice and sugar cane fields of the new agricultural zone, resulted in a spread of schistosomiasis (bilharzia) among human populations living or working upriver of the Diama dam [4], [5], [15]. Chemotherapy-based campaigns using praziquantel, the primary drug used today to fight schistosomiasis, have been carried out by the Senegalese government. However, to eliminate the disease, an integrated management program is required. While praziquantel effectively kills adult worms inside the definitive host's body, rapid reinfection can occur upon re-exposure to cercariae from infected snails in the environment. [16]–[18].

Snail population abundance and distribution are mediated by predators in several aquatic systems [19]–[22]. Accordingly, *Macrobrachium rosenbergii*, the most commonly aquacultured freshwater prawn in the world [23], is an effective predator of medically important freshwater snails under laboratory conditions [24]–[26]. Similarly, due to its relatively large size and tendency to consume medically important snails, *M. vollenhovenii* has been proposed both as a candidate for commercial aquaculture [27]–[29] and as an agent for biological control of schistosomiasis [25]. Indeed, *M. vollenhovenii* prawns were successful in controlling schistosome-susceptible snail populations under laboratory conditions [25]. Like other freshwater prawns, *M. vollenhovenii* exhibits clear sexual dimorphism, with males achieving larger maximum size than females [30]–[32]. Sexual dimorphism in many crustaceans is mediated by secretions of the androgenic gland (AG), a masculinizing endocrine organ unique to this sub-phylum [33]–[36]. The masculinity-regulating hormone secreted by this gland in decapod crustaceans is the insulin-like hormone of the androgenic gland (IAG). The gene encoding the hormone is uniquely expressed in males, with the function of the protein having been studied in several species [37]–[40]. Following discovery of the AG in *M. rosenbergii* [41], a full functional sex reversal was achieved by bilateral ablation of the gland [42], [43]. The discovery and sequence of the IAG-encoding gene in *M. rosenbergii* (*Mr-IAG*) [44] opened a path for the development of an innovative method of sex reversal through temporal RNA interference (RNAi) using double-stranded *Mr-IAG* RNA [42], [45]. In this manner, sex reversed males (neo –females) are created that, when crossed with normal males, produce all-male progeny. Since male prawns grow faster than females and reach a larger size, these findings were translated into a commercialized biotechnology, namely the first use of RNAi in aquaculture, initially applied for the production of all-male prawn populations [46]. We hypothesized that the same could be achieved with other *Macrobrachium* species, such as *M. vollenhovenii*.

In this multi-disciplinary study, we assessed the current abundance of *M. vollenhovenii* prawns in the Senegal River basin through capture using baited prawn traps. Such trapping efforts were supplemented by collaboration with local fisherman via a program offering purchase of their prawn catches throughout the course of the study period. We also conducted retrospective interviews with fishermen regarding the abundance of prawns along the Senegal River basin before and after construction of the Diama Dam. Studies of prawn catches and earlier literature on male superior size [31], [32] suggested that both prawn fisheries and their biological control functions could benefit from restocking with all-male populations. A further objective of this study was thus to lay the required molecular foundation for the production of all-male populations. Accordingly, we characterized the AG and the *IAG*-encoding gene of *M. vollenhovenii* as a first step towards producing all-male populations for mass restocking of biological control agents.

## Materials and Methods

### Monitoring prawn abundance in the Senegal River basin

**Figure 1:**
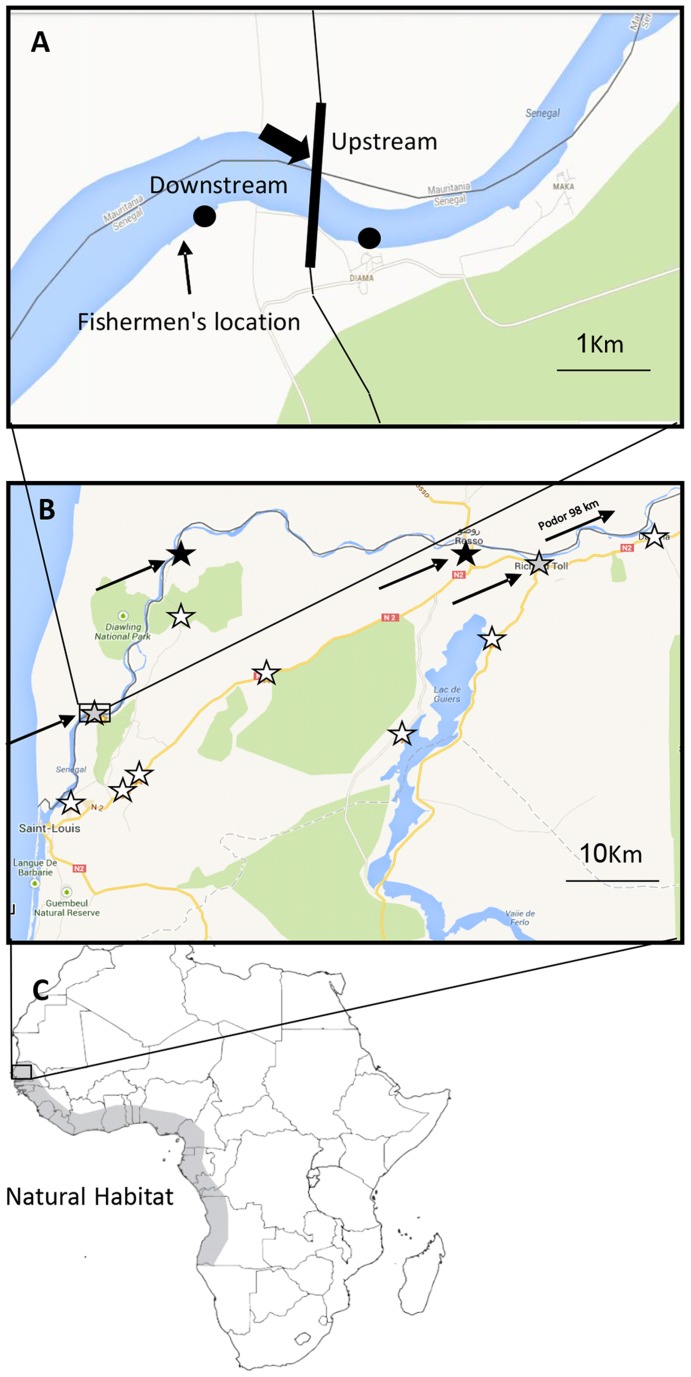
Project locations. Map of the Diama Dam area. (A) The Diama Dam (marked with a black arrow). The area in which the prawns were caught is marked “Fisherman's location”. Map of the Senegal River basin. (B). The areas of the survey are marked with stars; black stars represent interview locations while white stars represent trapping locations. Grey stars denote sites where both a trap was placed and an interview was conducted. (C) Map of Africa. The natural distribution area of *M. vollenhovenii* along the west coast of Africa is marked in gray.

To monitor the current abundance of prawns upstream of the Senegal River basin, 2–4 large crayfish traps were placed for 17–24 hours per site-visit at 15 sites throughout the lower Senegal River basin (Fig. 1B, marked with white and grey stars). Sites were visited bimonthly between February, 2011 and June, 2012. The traps used were commercial cylindrical crayfish traps constructed of a collapsible metal frame 30 cm in diameter and 60 cm in length, surrounded by fishing-net material. Traps were equipped with bait (either dead fish or meat plus vegetables, such as cassava root or local plant material, as recommended by local prawn fishermen). The traps and baits were tested in 9 m^2^ prawn tanks at the Senegalese National Aquaculture facility prior to deployment and were found to successfully capture prawns within a few hours.

To compare the abundance and distribution of prawns upstream of the Diama Dam in the Senegal River basin with abundance in the vicinity of the Diama Dam, prawns were purchased from local fisherman. All *M. vollenhovenii* prawns used for the present study were collected from September 12, 2012 to August 31, 2013 (excluding April and July, 2013, due to budgetary obstacles) by a group of six fishermen who work regularly both up- and downstream of the Diama Dam (Fig. 1A). All prawns caught by fisherman were captured near the Diama Dam in the Saint-Louis region, Senegal (N 16°12′52,65″ W 25°20′16,07″, marked as “Fisherman's location” in Fig. 1A). The fishermen used three types of fishing techniques, including baited traps (60 cm high, 80 cm diameter, made of metal and covered with fishing net), “sleeping nets” (200×6 m nylon net, 36 mm mesh with a 2 mm string) and a “drifting net” (same material as the sleeping net). The drifting nets are built of three nets attached together (600×6 m), so as to cover the width of the river.

Since little quantitative information on prawn abundance in the past was available for this study, attempts to compare current abundance with the situation before construction of the dam relied on retrospective interviews with fishermen in villages along the Senegal River (Fig. 1B, marked with black stars). This complementary approach included a standard questionnaire (see supplemental appended item S1) designed to solicit information on the prawn catch today, compared to the past. The fishermen were asked twenty questions, including verification of their fishing experience (years of activity) and whether fishing is their primary activity (in order to estimate their reliability). Fishermen were shown pictures of *M. vollenhovenii* to confirm or reject prior recollection of the prawns by appearance. Locations where both trapping and interviews were conducted are marked with grey stars on the map in Fig. 1B. Non-parametric statistical analysis was conducted to compare the reported abundance of the prawns before the construction of the dam and today in five villages upstream of the dam. Concomitant with the decline in prawn abundance, the number of active fishermen in the five villages, was reported by the fishermen to have declined from 175 before construction of the dam to only 18 today that were approached. Of these, the five who were active before construction of the dam and remain active today were selected for the study (one from each village).

### Statistical analysis of weight comparisons between males and females

To examine the relationship between sex and body weight, a two-sample Kolmogorov–Smirnov test, comparing the data distribution of both sexes, was initially conducted. An R×C test of independence was then performed to determine whether there was a dependency between sex and body weight, relying on the frequency of males and females weighing above 100 g. All analyses were conducted using STATISTICA 10 (StatSoft software, Tulsa, OK).

### Molecular studies

All Prawns used in the molecular study were anesthetized on ice for 5 min prior to dissection. Species determination was based on a molecular analysis using PCR for amplification of *M. vollenhovenii* mitochondrial 16S rRNA sequence (GenBank accession numbers see Table 1.). RNA samples from animals caught by the fishermen (see “Monitoring prawn abundance in the Senegal River basin”) were extracted and cDNA was prepared for PCR amplification as previously described [47]. For PCR amplification, the forward and reverse primers listed in Table 2 were used. PCR products were separated on agarose gels and bands were excised, purified and cloned as previously described. Sequences were obtained and compared to the known sequences using the BLAST algorithm.

**Table 1:** Gene Bank accession numbers.

Sequence title	organism	Accession number
mitochondrial 16S rRNA	*Macrobrachium vollenhovenii*	JQ943722.1
IAG	*Macrobrachium vollenhovenii*	KJ524578
*β*-actin	*Macrobrachium rosenbergii*	AF221096
IAG	*Macrobrachium rosenbergii*	FJ409645
IAG	*Portunus pelagicus*	HM459854
IAG	*Cherax quadricarinatus*	DQ851163
IAG	*Fenneropenaeus chinensis*	JQ388277.1
IAG	*Macrobrachium nipponense*	KC460325.1
IAG	*Penaeus monodon*	GU208677.1
IAG	*Callinectes sapidus*	HM594945.1
IAG	*Marsupenaeus japonicus*	AB598415
IAG	*Palaemon paucidens*	AB588013.1
IAG	*Palaemon pacificus*	AB588014
IAG	*Macrobrachium lar*	AB579012.1
IAG	*Cherax destructor*	EU718788
Insulin Protein	*Caenorhabditis elegans*	2KJI_A

**Table 2:** Primers used in the present study.

Primer use	Forward 5′ to 3′	Reverse 5′ to 3′
Species determination	CCGTGCGAAGGTAGCATAGTCAG	AACTCTCAAGGAAAATCACGCTG
RT-PCR	GACAGCGTGAGGAGAAGTCC	TATAGGACAGGGACGG GATG
RT-PCR positive control (*M. rosenbergii β*-actin)	GAGACCTTCAACACCCCAGC	AGGTGGTCTCGTGAATGCC
*Mv-IAG*	GTTCCTCTGCTCACTCGTAACACT	CTCCTCCTCCTCTTCCACCTTA
RACE	GAAGAAGCGAACAAGATGCTGCAAT	CTCTTTGGAAATGTAGGTGGGTCC

#### RT-PCR and *M. vollenhovenii* insulin-like androgenic gland hormone (Mv-IAG) tissue specificity

To enable easier identification of the AG, an endocrine manipulation of bilateral eyestalk ablation, causing hypertrophy of the AG (hAG), was performed on three mature males, as previously described [39]. Dissected AGs were placed in RNA SAVE (Biological Industries, Beit Haemek, Israel) and transported for molecular analysis at Ben-Gurion University, Beer-Sheva, Israel. RNA from the hAG of an endocrinologically-manipulated male caught by the fishermen (see “Monitoring prawn abundance in the Senegal River basin”, manipulation described above) was extracted and cDNA was prepared as described above. The cDNA was then amplified by PCR, as previously described [44], using specific forward (nt 627–646) and reverse (nt 770–789) primers based on the sequence of *Mr-IAG*. *M. rosenbergii β*-actin (Table 1) served as a positive control using appropriate forward and reverse primers. PCR products were cloned and sequenced.

Tissue specificity was determined by PCR using cDNA prepared from several mature animal tissues (AG, ovary and hepatopancreas), as described above. The cDNA was then amplified by PCR using specific *Mv-IAG* forward (nt 310–334) and reverse (nt 589–611) primers. *M. rosenbergii β*-actin served as a positive control [44]. All primers used in these studies are listed in Table 2.

#### Sequencing *Mv-IAG* and bioinformatics analysis

The sequences of the 5′ and 3′ ends of *Mv-IAG* were obtained by 5′ and 3′ rapid amplification of cDNA ends (RACE) using the SMARTer RACE kit (Clontech) following the manufacturer's protocol. PCR amplification of the 5′ region was achieved using the gene-specific reverse primer from the 3′ Race kit, (nt 108–132) and the Universal Primers Mix (UPM) provided with the kit. PCR amplification of the 3′ region was performed with the UPM as a reverse primer and the gene-specific forward primer (nt 297–322). The PCR products were cloned and sequenced as described above. Following determination of the full sequence of *Mv-IAG*, a multiple sequence alignment, including its deduced peptide, was conducted using the CLUSTAL W algorithm and IAG sequences from three representative species belonging to different crustacean families: *M.rosenbergii*, *Portunus pelagicus*, *Cherax quadricarinatus* and *Fenneropenaeus chinensis* (Table 1).

Phylogenetic analysis was conducted using MEGA, version 4.0 [48]. Such analysis considered eight additional crustacean species (listed in Table 1) and an insulin protein from *Caenorhabditis elegans* as an out-group. Evolutionary history was inferred using the Neighbor-Joining method [49]. The bootstrap consensus tree inferred from 5,000 replicates was taken as representing the evolutionary history of the selected mature IAGs among the taxa analyzed.

### Histology and immunohistochemistry

AGs were dissected from mature males, together with the attached terminal ampullae, under laboratory conditions in Senegal. Tissue samples were fixed in modified Carnoy's II for 72 h while being transported to Ben-Gurion University, Israel, where they were further processed according to conventional procedures. Five µm-thick sections were prepared. One out of five consecutive slides was stained by hematoxylin and eosin as previously described [47].The other four slides were analyzed by immunohistochemistry using rabbit α-rec-Mr-IAG antibodies (1∶1500) as previously described [50].

## Results

### Presence of *M. vollenhovenii* in the Senegal River basin

#### *M. vollenhovenii* trapping in the Senegal River basin

In the survey of current prawn abundance in the Senegal River basin, only three adult prawns (two of them *Macrobrachium vollenhovenii* and one *Atya* sp.) were trapped over a 16-month span corresponding to a total of 6,297 trap-hours of effort. The two *M. vollenhovenii* prawns were captured in the vicinity of Diama Dam, one upstream of the dam and the other downstream (black circles in Fig. 1A). In addition, juvenile *Macrobrachium* prawns (identified to genus only – adult specimens are required to identify species) were encountered below the dam, but not above. At the other thirteen upstream locations (white and grey stars in Fig. 1B), no *Macrobrachium* spp. prawns were captured despite 5,354 trap-hours of effort. A total of 359 fish, 40 crabs and one turtle were caught in traps during the same surveys.

#### Purchased catches of *M. vollenhovenii* around the Diama Dam and weight comparison between sexes

**Figure 2:**
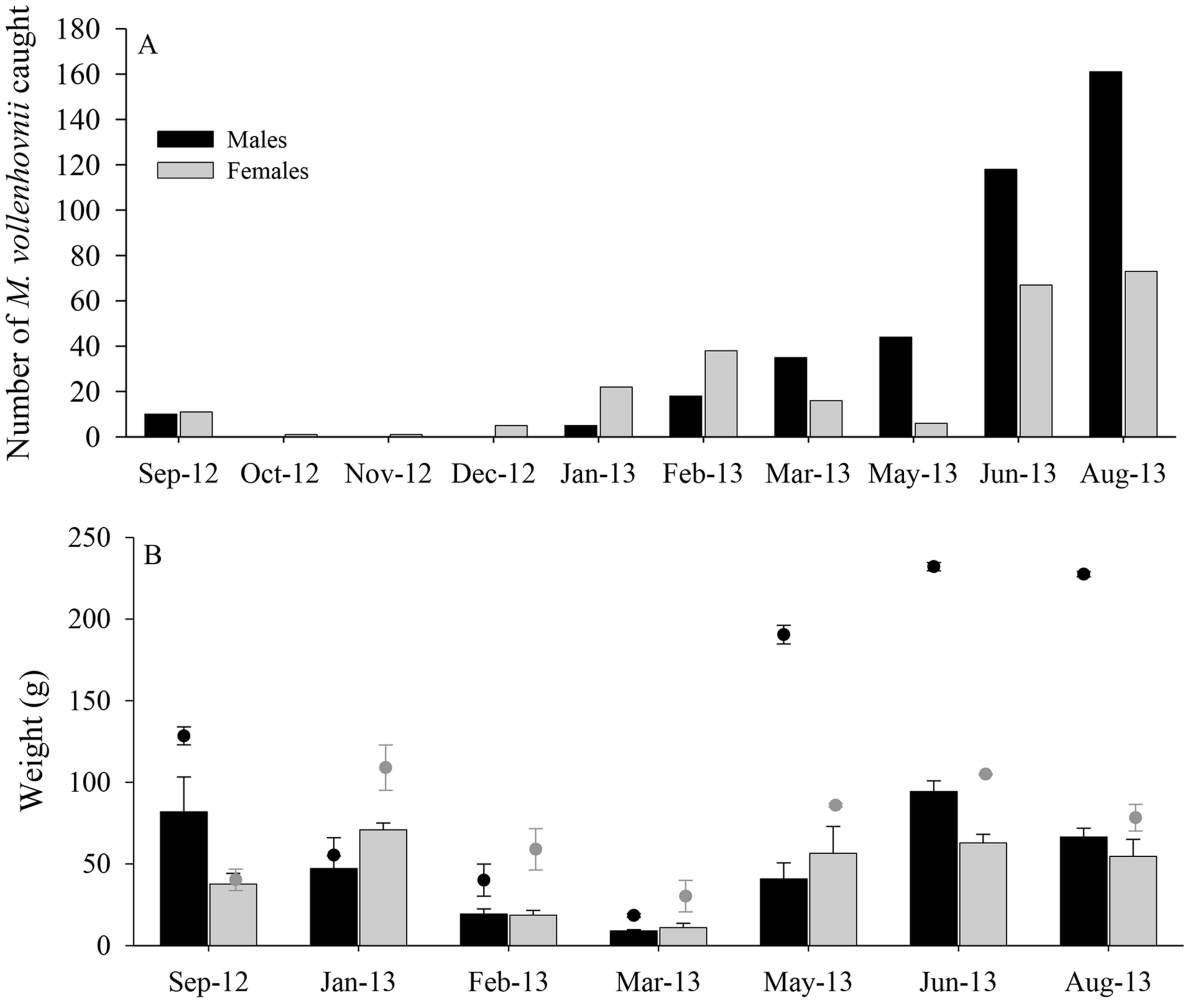
Monthly distribution of *M. vollenhovenii* catches in the Senegal River. (A) Total catch of 631 prawns around Diama Dam during 10 months between September, 2012 and August, 2013. (B) Comparisons between male and female average sizes and an average of the largest three specimens in each group. Bars represent SEM.

During the 12-month period of the survey, 631 *M. vollenhovenii* specimens were caught and supplied by fishermen from locations near the Diama Dam. A monthly distribution of males versus females is presented in Fig. 2A. The distribution of the prawns caught upstream or downstream of the dam varied throughout the year. According to fishermen, between June, 2012 and January, 2013, about 80% of the prawns were collected downstream of the dam, and 20% upstream, near Diama village (based on reported retrospective estimates relying on the fishermen's recall over the survey period, Fig. 1A). However, between February and May, 2013, the trend was the opposite, with 80% of the prawns reported to be caught upstream.

Comparing the average and maximal weights of males versus females (Fig. 2B) indicated that in most cases, the average weight of males was greater. More noteworthy were the maximal weights recorded from the three largest male or female specimens, which indicated that males could achieve a much larger maximal weight. Histograms depicting male and female weights show a bi-modal frequency distribution (Fig. S2) that divides the male population into two different weight groups, namely 0–100 and 100–240 g. When considering 100 g or more as a ‘large’ specimen, a significant pattern of sex dependency was found, with 17.7% of the males being ‘large’ versus 1.4% of the females (χ^2^_1_ = 20.07, P<0.001). Thus, our data suggest that males reach larger weights than females, especially during the wet season (June to September, reported as the best time to fish for prawns). The largest female encountered during the study weighed 129 g, as opposed to 235 g for the largest male.

#### Interview survey of fisherman from the Senegal River basin

Retrospective comparison of the current prawn abundance with the situation before dam construction reflected a dramatic decrease in the reported catch after the Diama Dam was constructed. Five fishermen from five different locations upstream of the Diama Dam (Fig. 1B) were approached with a retrospective questionnaire (see supplementary material S1). All claimed to have caught prawns routinely before the dam was built, whereas today only a very small prawn catch was claimed. If caught, the maximum quantity of prawns captured, as reported by the fishermen, was significantly lower than quantities reported from the time before construction of the dam (Table 3, T = 0.00, P = 0.04, Wilcoxon matched pairs test). All of those interviewed have been active fisherman for at least 45 years and are thus first-hand witnesses of the dam construction event.

**Table 3:** The quantity of prawns (in kg) caught at different locations in the Senegal River upstream of the Diama Dam during one week of fishing, as compared to numbers before construction of the dam, according to fishermen interviews.

REGION	MAX BEFORE-DAM CATCH	MIN BEFORE-DAM CATCH	MAX CATCH TODAY	MIN CATCH TODAY
**Diama**	2,000	1,000	20	5
**Debi**	80	60	1	0
**Rosso**	90	70	4	2
**Richard Toll**	100	80	5	2
**Podor**	80	70	3	1

### The full-length cDNA of *Mv-IAG*: Encoding sequence and deduced peptide, multiple sequence alignment with decapod IAGs and phylogenetic analysis

**Figure 3:**
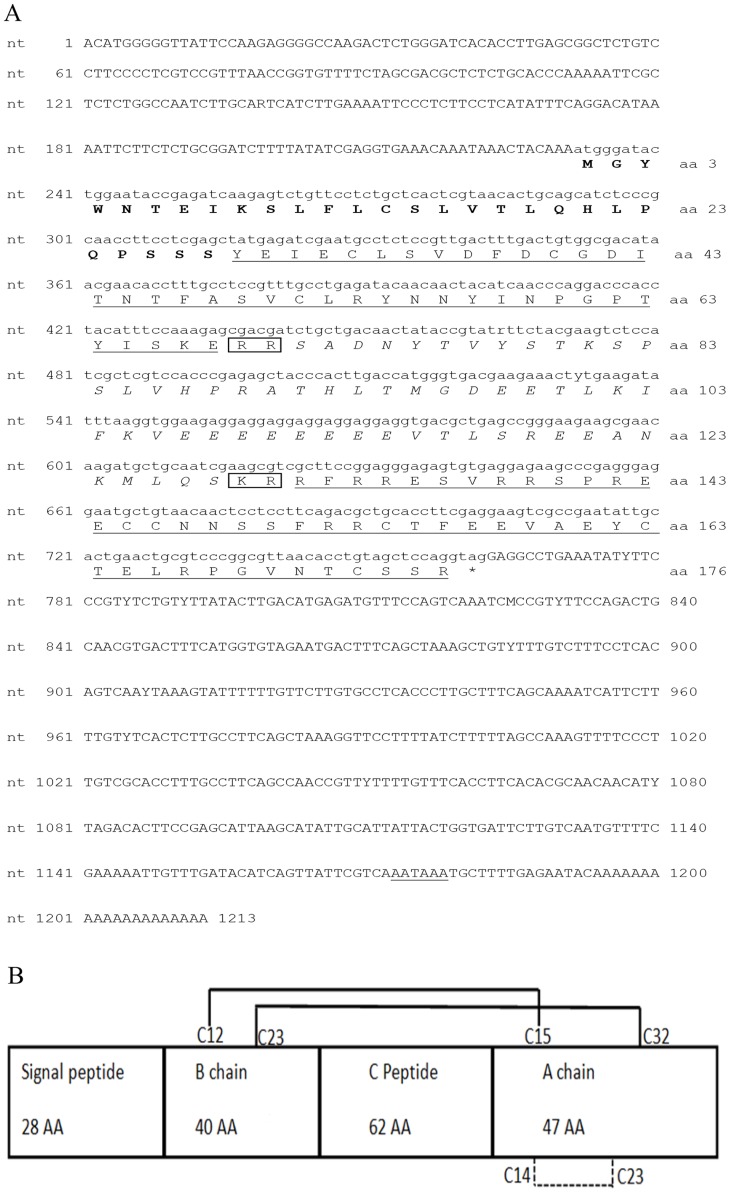
The *M. vollenhovenii IAG* gene and its deduced amino acid sequence. (A) *Mv-IAG* cDNA sequence and deduced Mv-IAG protein. The amino acids of the signal peptide (encoded by nucleotides 231 to 315) are shown in bold. The putative B and A chains are underlined and putative C peptide is *italicized*. The predicted arginine C-proteinase cleavage sites are boxed. The stop codon is mark with an asterisk. (B) Linear model of Mv-IAG. The model describes the deduced sequence of the components of prepro-Mv-IAG, the signal peptide, B chain, C peptide and A chain. The mature hormone consists of the B and A chains interlinked by two disulfide bridges; a third disulfide bridge, an intra-chain bridge, is formed within the A chain.

Due to the above size/weight differences found between *M. vollenhovenii* males and females and the notion that restocking with an all-male population will be advantageous, the AG and hormone that mediate maleness in this species were studied. Full-length *Mv-IAG* cDNA was found to be 1,213 bp-long (Fig. 3A, Accession number KJ524578). The sequence was isolated from a hAG by means of RT-PCR using *Mr-IAG*-based primers, followed by 5′ and 3′ RACE. The results showed that *Mv-IAG* consists of an open reading frame (ORF) of 531 bp flanked by a 5′ UTR (231 bp) and a 3′ UTR (451 bp) containing the putative polyadenylation site AATAAA. The *Mv-IAG* ORF was also predicted by ORF Finder (http://www.ncbi.nlm.nih.gov/gorf/gorf.html). A 28 amino acid-long signal peptide was predicted by SignalP (http://www.cbs.dtu.dk/services/SignalP).

The predicted *Mv-IAG* ORF encodes a preprohormone, a signal peptide, the B chain, the C peptide, and the A chain in linear order (Fig. 3B). The B and A chains of Mv-IAG are thought to be connected by two putative inter-chain disulfide bridges formed between Cys12 and Cys23 residues of the B chain and Cys15 and Cys32 of the A chain. Two other cysteine residues located in the A chain, Cys14 and Cys23, are suggested to form an intra-chain disulfide bridge. Two putative cleavage sites of RR and KR at amino acids 69 and 129, flanking the C peptide were joined to the B and A chains, respectively.

**Figure 4:**
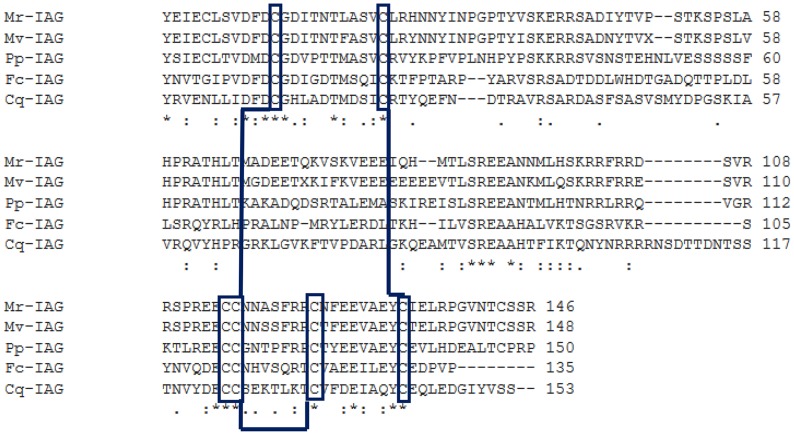
Multiple-sequence alignment of Mv-IAG with four IAGs of representative decapods from different groups (prawn, shrimp, crayfish and crab). Shown are Mr-IAG from *M. rosenbergii* (freshwater prawn), Pp-IAG from *Portunu spelagicus* (crab), Cq-IAG from *Cherax quadricarinatus* (crayfish) and Fc-IAG from *Fenneropenaeus chinensis* (marine shrimp). The sequences were aligned using the CLUSTAL W algorithm. The degree of conservation is presented by the dots under the columns. One dot represents less conserved than two dots, while an asterisk indicates identity. The most conserved feature is the backbone consisting of six cysteine residues (boxed) which gives rise to disulfide bridges (lines connecting the boxes).

**Figure 5:**
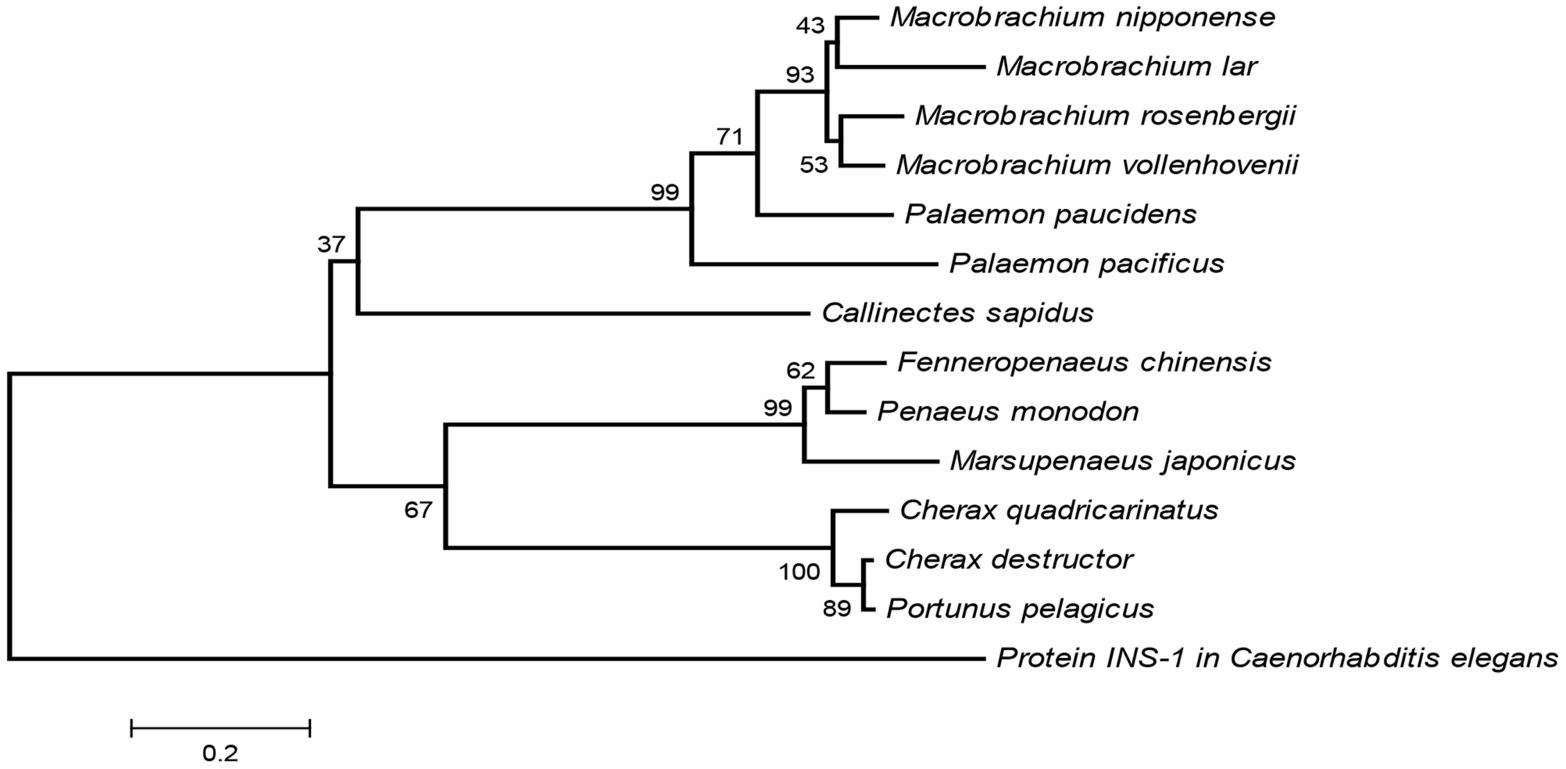
Phylogenetic tree of the IAGs. The tree is based on the CLUSTAL W algorithm of all known IAGs from decapod crustacean species, calculated and presented by MEGA4 [48]. A *C. elegans* insulin-like protein serves as an out-group. The numbers on the junctions represent the percentage of attempts, reflecting the specific divergence within 5,000 replicates, while the bar represents the number of amino acid substitutions per site.

The Mv-IAG sequence was compared with those from four other decapod crustacean species (*M. rosenbergii*, *P. pelagicus*, *C. quadricarinatus* and *F. chinensis*) in a multiple sequence alignment (Fig. 4). The positions of twenty amino acids were conserved. These included six cysteine residues, with two found in the B chain and four in the A chain. A phylogram generated using neighbor-joining methods [49] segregated the different decapod IAGs in accordance to their genus (Fig. 5). Protein INS-1 of *C. elegans* was used as an out-group to all of the twelve decapod IAGs known to date. It is clear that Mv-IAG is more related to Mr-IAG than to any other sequence. The different clades in the phylogram, reflecting the similarities of the proteins in the different species, were found to correlate with taxonomic relations in the cases of the *Macrobrachium*, the *Palaemon* and the *Cherax* species.

### Localization of the AG, Mv-IAG tissue specificity at the transcript and protein levels

**Figure 6:**
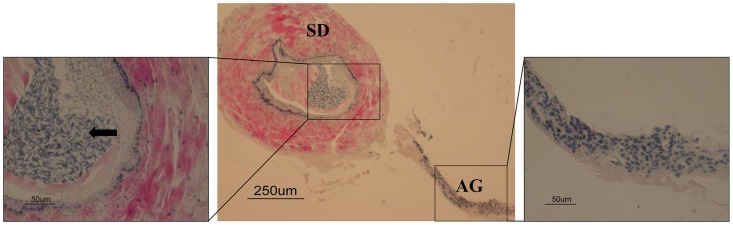
Histological sections of the sperm duct and AG of a mature *M. vollenhovenii* male stained with hematoxylin and eosin. The center picture shows the sperm duct (SD) and the androgenic gland (AG). A zoom section of the AG with a 50 µm bar and the nuclei of the cells can be seen (right). The left figure is a zoom of the sperm duct, where spermatozoa can be seen in the lumen (shown in arrow).

**Figure 7:**
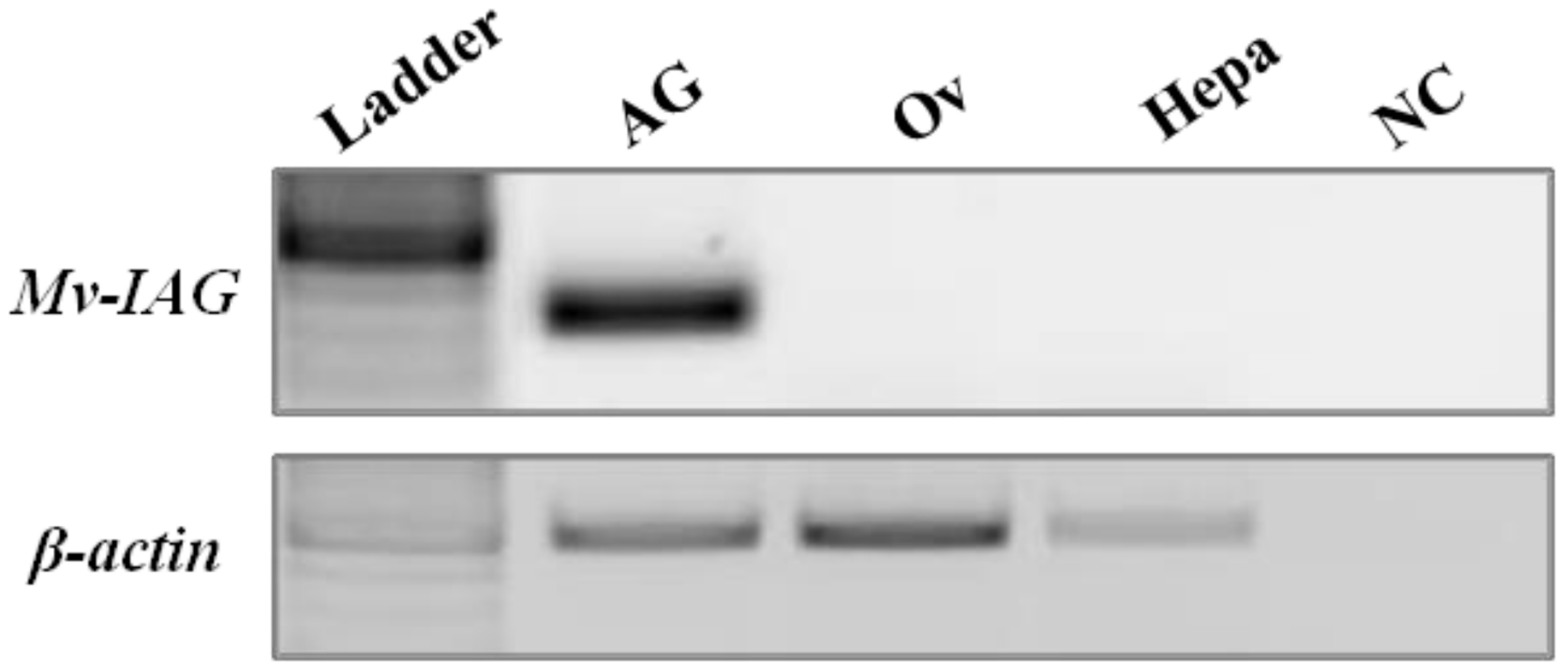
Demonstration of *Mv-IAG* transcription in the AG of a sexually mature *M. vollenhovenii* male. RT-PCR showed no amplification of this transcript in the ovary (Ov) of a female or in the hepatopancreas (Hepa) of a male. Transcription of *M. rosenbergii* β-actin (table 1) served as a positive control. A negative control (NC) contained no cDNA template.

The AG is located next to the sperm duct (Fig. 6 middle). The sperm duct wall is rich in muscle fibers and filled with mature spermatozoa (Fig. 6 left). *Mv-IAG* transcription was demonstrated by RT-PCR of cDNA from the AG but not from the male hepatopancreas or female ovary. The *M. rosenbergii* housekeeping gene *β-actin* served as a positive control (Fig. 7).

**Figure 8:**
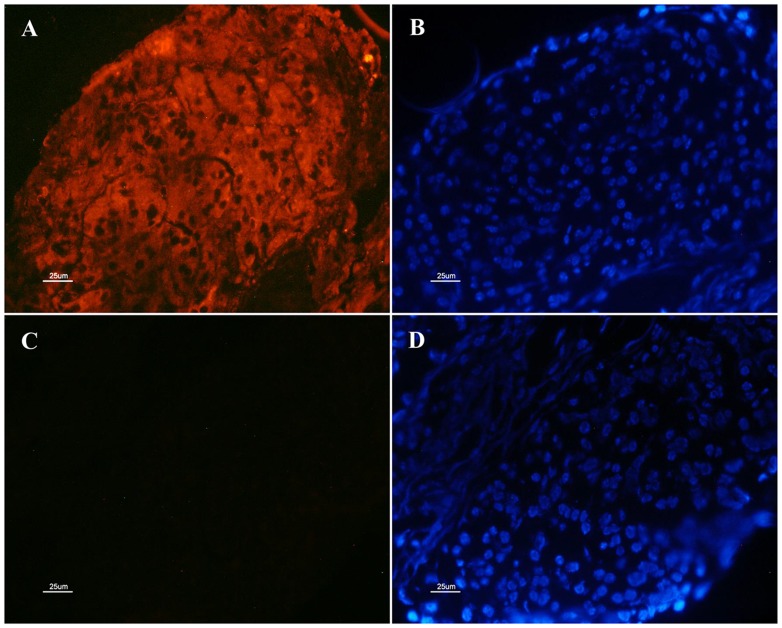
Immunohistochemical localization of Mv-IAG. The top pictures (A, B) show sections incubated with anti-*Mr-IAG* anti-serum, while the bottom pictures (C, D) portray controls incubated only with normal rabbit serum. The AG nuclei are stained blue with DAPI (B, D). A specific signal (stained red with Cy^3^) appears only in the cytoplasm of the treated AG cells (A, top left). No specific signal appears in the negative control sections incubated only with normal rabbit serum (C).

Based on immunohistochemical analysis, Mv-IAG was localized to hAGs (Fig. 8), using rabbit anti-Mr-IAG specific antibodies [50]. A specific signal was observed only in the cytoplasm of the AG cells (Fig. 8A), as nuclei were only stained by DAPI and not by the antibodies (Fig. 8B). The specificity of the anti-Mr-IAG antibodies was further validated when no signal could be observed upon incubation of normal rabbit serum with the AG sections (Fig. 8C). Sections were also stained with DAPI, which enabled nuclear localization as negative controls (Fig. 8D).

## Discussion

Early malacological literature suggests that the outbreak of schistosomiasis in the Senegal River basin occurred due to ecological changes resulting from the construction of the Diama and Manantali Dams, which were completed in 1986 and 1990, respectively [4], [6], [15]. Our current surveys in the Senegal River basin, including retrospective information from fishermen, appear to confirm the notion that the abundance of *M. vollenhovenii* was negatively influenced by construction of the Diama Dam. Although the historical, interview-based data could not be confirmed with independent fisheries or catch data prior to the appearance of the dam, research has consistently shown fishermen's knowledge to be a reliable estimate of relative abundance and distribution of fished species [51], [52]. Moreover, during the present study, fishermen in the Diama Dam region received an incentive to fish *M. vollenhovenii* in the form of a reward offered by the current project. This presented yet further evidence supporting the reduction in abundance reported by fishermen as these individuals now devoted considerable effort to the prawn catch. Still, despite the increased effort, the data collected were comparable to those reported in the interviews. However, the causal relationship between prawn scarcity and the increased abundance of the snails and schistosomiasis infections upriver of the Diama Dam could not be established using our correlative data and should be further investigated.

The use of prawns as biological control agents has been suggested and tested with both *M. rosenbergii* and *M. vollenhovenii*, showing that freshwater prawns are effective predators of schistosome-susceptible snails under laboratory conditions [24]–[26]. The novel approach of restocking populations of an indigenous prawn for its biological control abilities could become a powerful complement to chemotherapy campaigns. Today, campaigns for the distribution of this drug focus on periodic administration of the anthelminthic, praziquantel, to kill the adult worms [53]. What is lacking is a sustainable control strategy to prevent re-infection from snail to man [16], [54]. The ability of an invasive, non-native crustacean to eliminate snails was shown in Kenya, with a concomitant reduction of prevalence and intensity of urinary schistosomiasis in school children [55]. To the best of our knowledge *M. vollenhovenii* is the first indigenous crustacean-predator proposed for such purposes. Our study suggests a strategy of restocking all-male prawns at a significant scale in the Senegal River basin involving a population that could be bred, hatched, and nurtured to the post-larval or juvenile stage in aquaculture facilities and then released into schistosomiasis transmission foci. If all-male prawn populations show an advantage in terms of yield and biological control effectiveness in the field, this strategy could have broad application in West African public health, fisheries and aquaculture sectors. Moreover, RNAi has been demonstrated to be a potent method for temporal gene manipulation in crustaceans [56] and indeed, a sexual shift has been achieved in all cases of IAG RNAi in crustaceans tested thus far [45], [57]. Furthermore, the present study shows the high similarity between the IAG of the African prawn and that of other species, including that species in which RNAi has been successfully performed. Results of the present study also suggest that male *M. vollenhovenii* prawns reach larger sizes than females, as has been reported in the past [31]. Thus, the strategy of monosex culture could prove advantageous, similar to the proven production advantages of such cultures in *M. rosenbergii* aquaculture. These proven aquaculture benefits include the faster growth rate of males [13], [43], [58], the ability to selectively harvest non-growing large males in order to stimulate a growth spurt in the subordinate morophtypes [59]–[61], and the premium market prices acquired by large specimens [30], [62]. All of these advantages also apply to the sustainable restocking of prawns for biological control of snails. Because there is a need to ensure that the prawns will feed within specific, snail-infested sites, it is logical to use all-male non-migrating agents, as suggested with other *Macrobrachium* species [12], [14].

The sustainability of the solution proposed here will depend on a fisheries policy encouraging the harvesting or culling of the largest dominant males in order to boost the growth of smaller males and to maximize yields, as is routinely done in prawn aquaculture [63]. Such a policy will enable avoidance of over-population of the river since the size of the population will depend on the ratio between stocking and fishing rates. Moreover, since different-sized prawns have been found to be differentially efficient in snail predation [25], a continuous restocking with younger, fast-growing male prawns will also support the biological control task.

To achieve the all-male cohorts desired for restocking and fisheries, the current biotechnology relies on molecular manipulation of the IAG [46]. Here, we characterize *M. vollenhovenii* AG and IAG as a first step towards the ultimate goal of enabling routine, all-male *M. vollenhovenii* culture via recently established temporal RNAi-based biotechnology [46]. *M. vollenhovenii* IAG, has been completely sequenced in the present study and was found to share high similarity with homologous molecules in other decapod crustaceans [44], [47], [64]. Mv-IAG contains all the components of an insulin family member [38]. The *M. vollenhovenii* AG is anatomically and histologically similar to that described in *M. rosenbergii* [44]. Of the known decapod IAGs, Mv-IAG had the highest similarity to the IAG of its congener, *M. rosenbergii* (85% identity). Immunohistochemical analysis using anti-Mr-IAG anti-serum demonstrated the presence of Mv-IAG in the cytoplasm of AG cells. The high sequence similarity of Mv-IAG and Mr-IAG, as shown by bioinformatics tools in this study, provided the lead to pursue what turned out to be a successful use of anti-Mr-IAG antibodies to localize Mv-IAG in immunohistochemistry.

Based on our results and the high similarity of *M. vollenhovenii* to *M. rosenbergii*, it is realistic to assume that the biotechnology proven to be effective for mass production of *M. rosenbergii* all-male populations in prawn aquaculture [46] can be directly implemented to the production of all-male *M. vollenhovenii* populations.

At least 90% of the 243 million people currently infected with schistosomiasis in the world are in Africa [1] and at least 100 million of the more than 700 million people at risk of infection reside in areas that experienced major water management manipulations (i.e. dams and irrigation schemes), as was the case in the Senegal River basin [4], [7]. A meta-analysis [7] found that schistosomiasis risk in Africa was doubled for people living near dams and irrigation schemes, compared with people far from these schemes. Our suggested sustainable model of control, namely restocking native all-male prawn populations in the Senegal River using aquaculture and biotechnology, both as biological control agents and as an augmented fisheries crop, if proven successful locally, could be useful at other locations throughout the west coast of Africa where *M. vollenhovenii* is native (Fig. 1C) and where they may have been recently extirpated by dams. It is noteworthy that the use of all-male populations could permit responsible and sustainable restocking in other regions of Africa where these prawns are non-native, given that they have little invasion risk because the all-male prawns cannot revert to females and, therefore, cannot reproduce.

## Supporting Information

Figure S1 **Questionare.** All fishermen were approached with the french version of the questionaire.
(DOCX)

Figure S2 **Frequency histograms of the weight distribution of females and males.** All 436 animals that were weighted during the survey period are presented in the histograms.
(DOCX)

Figure S3 **Test of dependency between sex and weight.** R * C test of dependency of all weighted animals during the survey period. Above 100 gram animals were considered “Large.”
(DOCX)

## References

[1] WHO (2013) Schistosomiasis - facts sheet. World health organization.

[2] OMVS/SOGED (2006) Rôle et enjeux du barrage de Diama dans la problématique du développement du bassin du fleuve Sénégal Dakar: Organisation pour la mise en valeur du fleuve Senegal (OMVS) la société de gestion et d'exploitation du barrage de Diama (SOGED). 22 p.

[3] Southgate V, Tchuem Tchuente LA, Sene M, De Clercq D, Theron A, et al (2001) Studies on the biology of schistosomiasis with emphasis on the Senegal river basin. Memorias do Instituto Oswaldo Cruz 96 Suppl: 75–78. 11586429 10.1590/s0074-02762001000900010

[4] Southgate VR (1997) Schistosomiasis in the Senegal River Basin: before and after the construction of the dams at Diama, Senegal and Manantali, Mali and future prospects. Journal of Helminthology 71: 125–132. 9192711 10.1017/s0022149x00015790

[5] Sow S, de Vlas SJ, Engels D, Gryseels B (2002) Water-related disease patterns before and after the construction of the Diama dam in northern Senegal. Ann Trop Med Parasitol 96: 575–586. 12396320 10.1179/000349802125001636

[6] Diaw OT, Vassiliades G, Seye M, Sarr Y (1991) Epidemiology of Intestinal Schistosomiasis with *Schistosomam mansoni* in Richard-Toll (Delta of Senegal River) - Malacological Survey. Bulletin de la Société de Pathologie Exotique 84: 174–183. 1914048

[7] Steinmann P, Keiser J, Bos R, Tanner M, Utzinger J (2006) Schistosomiasis and water resources development: systematic review, meta-analysis, and estimates of people at risk. Lancet Infect Dis 6: 411–425. 16790382 10.1016/S1473-3099(06)70521-7

[8] Paterson JR (2007) The Kunene river mouth: managing a unique environment: University of KwaZulu-Natal. 112 p.

[9] Willfuhr-Nast J, Rosenthal H, Udo PJ, Nast F (1993) Laboratory cultivation and experimental studies of salinity effects on larval development in the African River prawn *Macrobrachium vollenhovenii* (Decapoda, Palaemonidae). Aquatic Living Resources 6: 115–137.

[10] Holthuis LB (1980) Shrimps and prawns of the world: an annotated catalogue of species of interest to fisheries. Rome: Food and Agriculture Organization of the United Nations. xvii, 271 p.

[11] GFCC (1980) Assessment and plan of action. Assessment of environmental effects of proposed developments in the Senegal river basin. Organisation pour la Mise en Valeur du Fleuve Senegal (OMVS). 246 p.

[12] Bauer RT (2011) Amphidromy and migrations of freshwater shrimps. ii. delivery of hatching larvae to the sea, return juvenile upstream migration, and human impacts. In: Asakura A, editor. New Frontiers in Crustacean Biology. Leiden: Koninklijke Brill NV. pp. 157–168.

[13] New MB, Singholka S (1982) Freshwater prawn farming. Rome: FAO. 116 p.

[14] Ling SW (1969) The general biology and development of *Macrobrachium rosenbergii* (De Man). FAO. 589–606 p.

[15] Talla I, Kongs A, Verle P, Belot J, Sarr S, et al (1990) Outbreak of intestinal schistosomiasis in the Senegal River Basin. Annales de la Societe belge de medecine tropicale 70: 173–180. 2122819

[16] Tchuem Tchuenté L-A, Momo SC, Stothard JR, Rollinson D (2013) Efficacy of praziquantel and reinfection patterns in single and mixed infection foci for intestinal and urogenital schistosomiasis in Cameroon. Acta Tropica 128: 275–283. 23791803 10.1016/j.actatropica.2013.06.007

[17] King CH, Sturrock RF, Kariuki HC, Hamburger J (2006) Transmission control for schistosomiasis - why it matters now. Trends Parasitol 22: 575–582. 17030017 10.1016/j.pt.2006.09.006

[18] Webster BL, Diaw OT, Seye MM, Faye DS, Stothard JR, et al (2013) Praziquantel treatment of school children from single and mixed infection foci of intestinal and urogenital schistosomiasis along the Senegal River Basin: monitoring treatment success and re-infection patterns. Acta Tropica 128: 292–302. 23022016 10.1016/j.actatropica.2012.09.010

[19] Bronmark C, Klosiewski S, Stein R (1992) Indirect effects of predation in a freshwater, benthic food chain. Ecology 73: 1662–1674.

[20] Hershey A (1990) Snail populations in arctic lakes: competition mediated by predation? Oecologia 82: 26–32. 28313133 10.1007/BF00318529

[21] Turner AM, Chislock MF (2007) Dragonfly predators influence biomass and density of pond snails. Oecologia 153: 407–415. 17457617 10.1007/s00442-007-0736-9

[22] Yamanishi Y, Yoshida K, Fujimori N, Yusa Y (2012) Predator-driven biotic resistance and propagule pressure regulate the invasive apple snail *Pomacea canaliculata* in Japan. Biological Invasions 14: 1343–1352.

[23] New MB, Tidwell JH, D'Abramo LR, Kutty MN (2009) Freshwater prawns: Biology and farming: John Wiley & Sons.

[24] Roberts JK, Kuris AM (1990) Predation and control of laboratory populations of the snail Biomphalaria glabrata by the freshwater prawn *Macrobrachium rosenbergii* . Annals of Tropical Medicine and Parasitology 84: 401–412. 2260905 10.1080/00034983.1990.11812486

[25] Sokolow SH, Lafferty KD, Kuris AM (2013) Regulation of laboratory populations of snails (*Biomphalaria and Bulinus spp*.) by river prawns, *Macrobrachium spp*. (Decapoda, Palaemonidae): Implications for control of schistosomiasis. Acta Tropica 132C: 64–67. 10.1016/j.actatropica.2013.12.013 PMC4280914 24388955

[26] Lee PG, Rodrick GE, Sodeman WA Jr, Blake NJ (1982) The giant Malaysian prawn, Macrobrachium rosenbergii, a potental predator for controlling the spread of schistosome vector snails in fish ponds. Aquaculture 28: 293–301.

[27] Brown JH (2006) Potential for shrimp culture in West Africa. Organisation for economic co-operation and development (OECD) Sahel and West Africa Club. 26 p.

[28] Marioghae IE (1988) *M. vollenhovenii:* guidelines for the implementation of the proposed pilot culture programme, making use of available infrastructure. Agege: Lagos State Agriculture Development Authority

[29] Holthuis LB (1980) FAO species catalogue. Volume 1-Shrimps and prawns of the world. An annotated catalogue of species of interest to fisheries. Rome: FAO.

[30] Sagi A, Raanan Z, Cohen D, Wax Y (1986) Production of *Macrobrachium rosenbergii* in monosex populations: yield characteristics under intensive monoculture conditions in cages. Aquaculture 51: 265–275.

[31] Marioghae IE, Ayilna OA (1995) The reproductive biology and culture of *Macrobrachium vollenhovenii* and *Macrobrachium macrobrachion* in Nigeria. Port Harcourt: Nigerian Institute for Oceanography and Marine Research

[32] Olele N, Kalayolo P (2012) Morphometric charectaristics of the Giant African River prawn, *Macrobrachium vollenhovenii* (Herklot, 1857) caught from Warri River coast. Journal of Agriculture and Biological Sciences 3: 232–239.

[33] Charniaux-Cotton H (1962) Androgenic gland of crustaceans. General and Comparative Endocrinology 1: 241–247. 13878306 10.1016/0016-6480(62)90095-3

[34] Katakura Y (1989) Endocrine and Genetic-Control of Sex-Differentiation in the Malacostracan Crustacea. Invertebrate Reproduction & Development 16: 177–182.

[35] Sagi A (1988) The androgenic gland in crustacea - with emphasis on the cultured freshwater Prawn *Macrobrachium rosenbergii* - A review. Israeli Journal of Aquaculture - Bamidgeh 40: 9–16.

[36] Sagi A, Snir E, Khalaila I (1997) Sexual differentiation in decapod crustaceans: Role of the androgenic gland. Invertebrate Reproduction and Development 31: 55–61.

[37] Lee T, Shigesawa R, Yamazaki F (1993) Partial masculinization of female *Eriocheir japonicus* (Brachyura, Grapsidae) by androgenic gland implantation. Suisanzoshoku 41.

[38] Ventura T, Rosen O, Sagi A (2011) From the discovery of the crustacean androgenic gland to the insulin-like hormone in six decades. General and Comparative Endocrinology 173: 381–388. 21679714 10.1016/j.ygcen.2011.05.018

[39] Khalaila I, Katz T, Abdu U, Yehezkel G, Sagi A (2001) Effects of implantation of hypertrophied androgenic glands on sexual characters and physiology of the reproductive system in the female red claw crayfish, *Cherax quadricarinatus* . General and Comparative Endocrinology 121: 242–249. 11254366 10.1006/gcen.2001.7607

[40] Cui Z, Liu H, Lo TS, Chu KH (2005) Inhibitory effects of the androgenic gland on ovarian development in the mud crab *Scylla paramamosain* . Comparative Biochemistry and Physiology, Part A: Molecular & Integrative Physiology 140: 343–348. 10.1016/j.cbpb.2005.01.017 15792600

[41] Nagamine C, Knight AW, Maggenti A, Paxman G (1980) Effects of androgenic gland ablation on male primary and secondary sexual characteristics in the Malaysian prawn, *Macrobrachium rosenbergii* (de Man) (Decapoda, Palaemonidae), with first evidence of induced feminization in a nonhermaphroditic decapod. General and Comparative Endocrinology 41: 423–441. 7409450 10.1016/0016-6480(80)90048-9

[42] Sagi A, Cohen D (1990) Growth, maturation and progeny of sex-reversed *Macrobrachium rosenbergii* males. World Aquaculture 21: 87–90.

[43] Aflalo ED, Hoang TTT, Nguyen VH, Lam Q, Nguyen DM, et al (2006) A novel two-step procedure for mass production of all-male populations of the giant freshwater prawn *Macrobrachium rosenbergii* . Aquaculture 256: 468–478.

[44] Ventura T, Manor R, Aflalo ED, Weil S, Raviv S, et al (2009) Temporal silencing of an androgenic gland-specific insulin-like gene affecting phenotypical gender differences and spermatogenesis. Endocrinology 150: 1278–1286. 18988670 10.1210/en.2008-0906

[45] Ventura T, Manor R, Aflalo ED, Weil S, Rosen O, et al (2012) Timing sexual differentiation: full functional sex reversal achieved through silencing of a single insulin-like gene in the prawn, *Macrobrachium rosenbergii* . Biology of Reproduction 86: 6. 10.1095/biolreprod.111.097261 22133694

[46] Ventura T, Sagi A (2012) The insulin-like androgenic gland hormone in crustaceans: From a single gene silencing to a wide array of sexual manipulation-based biotechnologies. Biotechnology Advances 30: 1543–1550. 22561950 10.1016/j.biotechadv.2012.04.008

[47] Manor R, Weil S, Oren S, Glazer L, Aflalo ED, et al (2007) Insulin and gender: an insulin-like gene expressed exclusively in the androgenic gland of the male crayfish. General and Comparative Endocrinology 150: 326–336. 17094989 10.1016/j.ygcen.2006.09.006

[48] Tamura K, Dudley J, Nei M, Kumar S (2007) MEGA4: Molecular Evolutionary Genetics Analysis (MEGA) software version 4.0. Molecular Biology and Evolution 24: 1596–1599. 17488738 10.1093/molbev/msm092

[49] Saitou N, Nei M (1987) The neighbor-joining method: a new method for reconstructing phylogenetic trees. Molecular Biology and Evolution 4: 406–425. 3447015 10.1093/oxfordjournals.molbev.a040454

[50] Ventura T, Manor R, Aflalo ED, Weil S, Khalaila I, et al (2011) Expression of an androgenic-gland-specific insulin-like peptide during the course of prawn sexual and morphotypic differentiation. ISRN Endocrinology 2011: 11. 10.5402/2011/476283 PMC3262648 22363879

[51] Poizat G, Baran E (1997) Fishermen's knowledge as background information in tropical fish ecology: a quantitative comparison with fish sampling results. Environmental Biology of Fishes 50: 435–449.

[52] Silvano RAM, Valbo-Jorgensen J (2008) Beyond fishermen's tales: contributions of fishers' local ecological knowledge to fish ecology and fisheries management. Environment Development and Sustainability 10: 657–675.

[53] WHO (2006) Preventive chemotherapy in human helminthiasis: coordinated use of anthelminthic drugs in control interventions: a manual for health professionals and programme managers. In: WHO, editor. Geneva. pp. 74.

[54] Bergquist R (2013) Closing in on ‘perhaps the most dreadful of the remaining plagues’: an independent view of the multidisciplinary alliance to optimize schistosomiasis control in Africa. Acta Tropica 128: 179–181. 23994520 10.1016/j.actatropica.2013.08.016

[55] Mkoji GM, Hofkin BV, Kuris AM, Stewart-Oaten A, Mungai BN, et al (1999) Impact of the crayfish *Procambarus clarkii* on *Schistosoma haematobium* transmission in Kenya. The American Journal of Tropical Medicine and Hygiene 61: 751–759. 10586907 10.4269/ajtmh.1999.61.751

[56] Sagi A, Manor R, Ventura T (2013) Gene Silencing in Crustaceans: From Basic Research to Biotechnologies. Genes 4: 620–645. 24705266 10.3390/genes4040620 PMC3927571

[57] Rosen O, Manor R, Weil S, Gafni O, Linial A, et al (2010) A Sexual Shift Induced by Silencing of a Single Insulin-Like Gene in Crayfish: Ovarian Upregulation and Testicular Degeneration. Plos One 5: e15281. 21151555 10.1371/journal.pone.0015281 PMC3000327

[58] Sagi A, Aflalo ED (2005) The androgenic gland and monosex culture of freshwater prawn *Macrobrachium rosenbergii* (De Man): a biotechnological perspective. Aquaculture Research 36: 231–237.

[59] Ra'anan Z, Sagi A (1985) Alternative mating strategies in male morphotypes of the freshwater prawn *Macrobrachium rosenbergii* (De Man). Biological Bulletin 169: 592–601.

[60] Fujimura T, Okamoto H (1972) Notes on progress made in developing a mass culturing technique for *Macrobrachium rosenbergii* in Hawaii. In: Pillay TVR, editor. Coastal Aquaculture in the Indo-Pacific Region. Fishing News Books: Blackwell Science, Oxford, UK. pp. 313–327.

[61] Cohen D, Sagi A, Ra'anan Z, Zohar G (1988) The production of *Macrobrachium rosenbergii* in monosex populations: III. Yield characteristics under intensive monoculture conditions in earthen ponds. Israeli Journal of Aquaculture 40: 57–63.

[62] Hulata G, Karplus I, Wohlfarth GW, Halevy A, Cohen D, et al (1988) The production of *Macrobrachium rosenbergii* in monosex populations. II. Yield characteristics in polyculture ponds. Israeli Journal of Aquaculture 40: 9–16.

[63] Rahman SMS, Wahab MA, Islam MA, Kunda M, Azim ME (2010) Effects of selective harvesting and claw ablation of all-male freshwater prawn (*Macrobrachium rosenbergii*) on water quality, production and economics in polyculture ponds. Aquaculture Research 41: e404–e417.

[64] Chung JS, Manor R, Sagi A (2011) Cloning of an insulin-like androgenic gland factor (IAG) from the blue crab, Callinectes sapidus: implications for eyestalk regulation of IAG expression. General and Comparative Endocrinology 173: 4–10. 21596044 10.1016/j.ygcen.2011.04.017

