# Supplementary material for: The Prawn Macrobrachium vollenhovenii in the Senegal River Basin: Towards Sustainable Restocking of All-Male Populations for Biological Control of Schistosomiasis
Source: PLoS Negl Trop Dis. 2014 Aug 28;8(8):e3060. doi: 10.1371/journal.pntd.0003060 (PMC4148216; doi:10.1371/journal.pntd.0003060)
Supplement: Figure S1 — Questionare. All fishermen were approached with the french version of the questionaire. (DOCX) [file pntd.0003060.s001.docx]

**Figure S1: questionnaire**

**Questionnaire on fresh water prawn *Macrobrachium vollenhovenii***

(Translated from French) –

**І. Introduction**

- Study presentation and objectives
- Importance of the interview
- Pictures of *M.vollenhovenii*

**ІІ. Interview**

1 – When did you begin to fish (year) ?

2 –Do you have other activities apart from fishing?

Yes No

3– If yes, which?

4 – What are the species that you fish?

Fish Shrimp Prawn

5 – What methods do you use to catch this/these species?

Fish

Shrimp

Prawn

6 – Capture frequency and fishing effort?

| Species | Fishing zone | Optimum capture frequency | | | Best fishing period  (month and time of the day) |
| --- | --- | --- | --- | --- | --- |
|  |  | Season | Month | Daily variation |  |
| Fish | Z1  Z2  Z3 |  |  |  |  |
| Shrimp | Z1  Z2  Z3 |  |  |  |  |
| Prawn | Z1  Z2  Z3 |  |  |  |  |

7 –What is the local name of this prawn (*M.vollenhovenii)* ?

8 – What is its peculiarity?

9 – Do you know how to distinguish between different species of prawns?

Yes No

10 – If yes, what differences are clearly noted?

11 – In which zone (see question 6) do you capture most of *M.vollenhovenii* in your area?

12 – What quantity of *M.vollenhovenii* could were you capturing (kg/day)?

- Before 1990
- From 1990 to 2000
- Since 2000

13 – Among thess 3 sizes, which is most frequent (a-small, b-medium, c-large)?

a /10 b /10 c /10

14 - Can you distinguish between the male and the female?

Yes No

15- If yes, what is the ratio? (M/F)

16- Can you distinguish between gravid and non-gravid females?

Yes No

17- If yes, what is the ratio? (Gravid/Non-gravid)

18- Currently, are you able to supply living specimens?

19- If yes, how would you catch them?

20- Do you know when was the Diama Dam was built?

**ІІІ. Personal details :**

**Name :**

**Phone**:

**Address** :
