# Supplementary figures and images for: The Prawn Macrobrachium vollenhovenii in the Senegal River Basin: Towards Sustainable Restocking of All-Male Populations for Biological Control of Schistosomiasis
Source: PLoS Negl Trop Dis. 2014 Aug 28;8(8):e3060. doi: 10.1371/journal.pntd.0003060 (PMC4148216; doi:10.1371/journal.pntd.0003060)

# Figure S2 – Frequency histograms of the weight distribution of females and males

Females


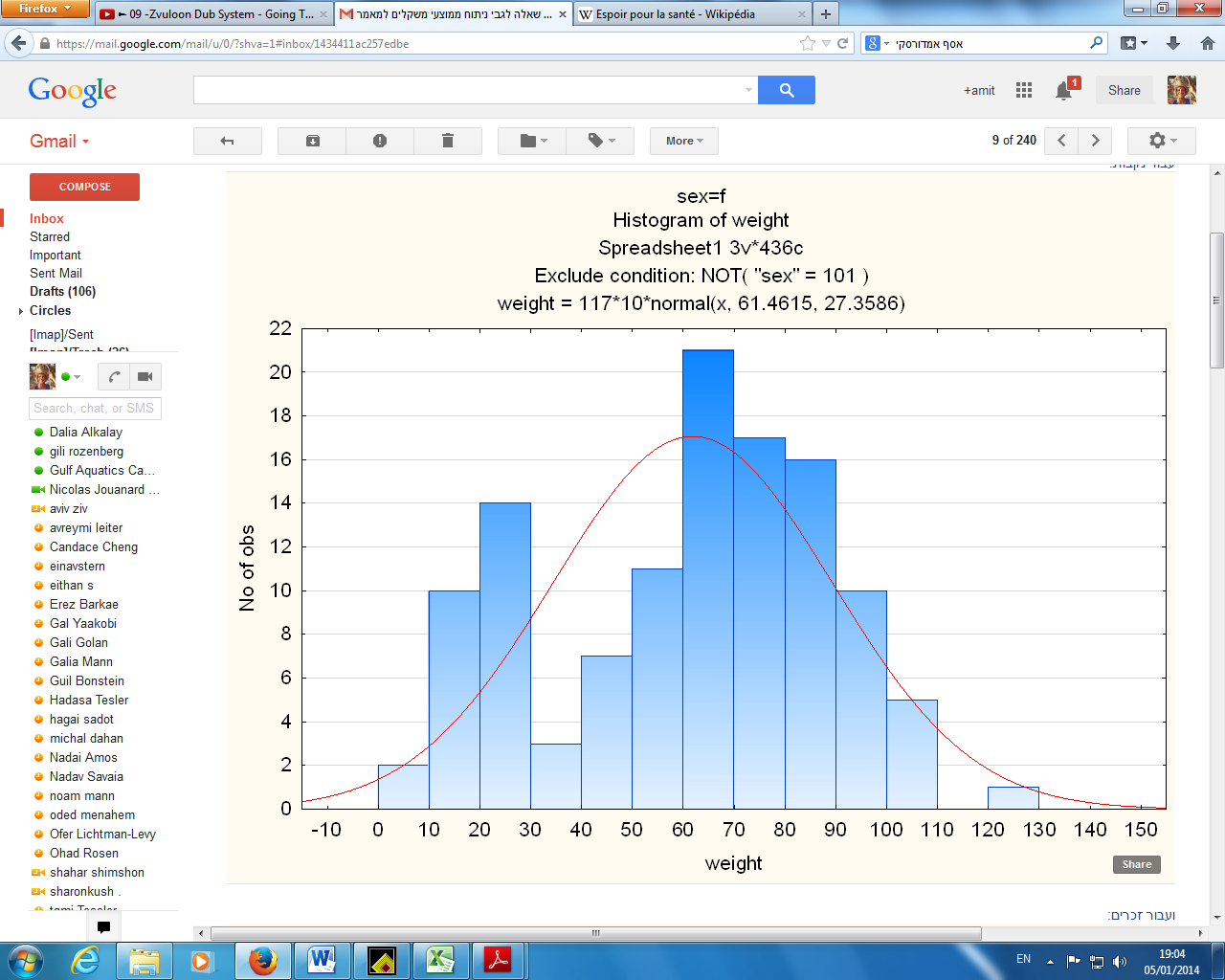


Males


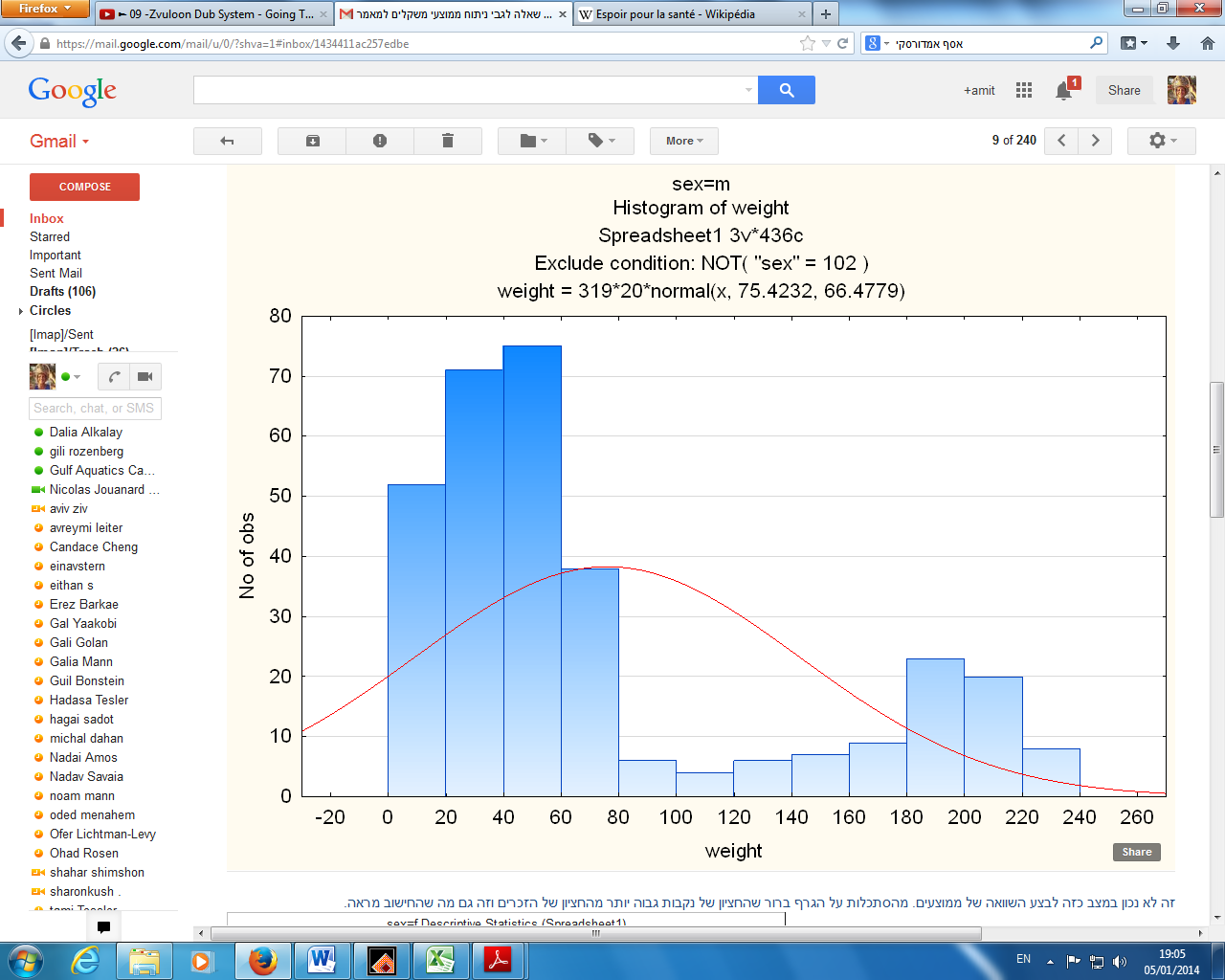

Supplement: Figure S2 — Frequency histograms of the weight distribution of females and males. All 436 animals that were weighted during the survey period are presented in the histograms. (DOCX) [file pntd.0003060.s002.docx]

**Figure S3. R * C test of dependency**


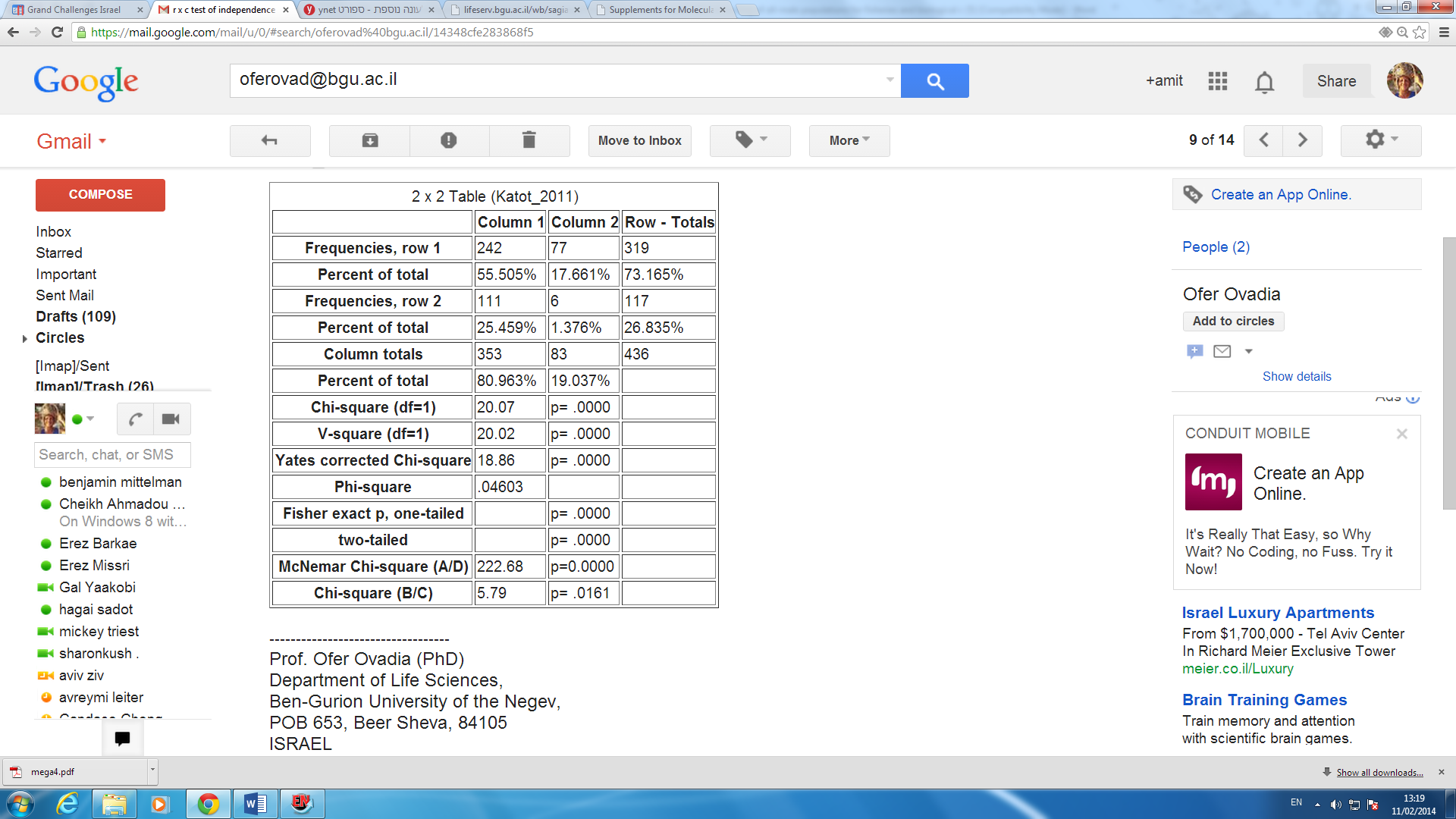

Supplement: Figure S3 — Test of dependency between sex and weight. R * C test of dependency of all weighted animals during the survey period. Above 100 gram animals were considered “Large.” (DOCX) [file pntd.0003060.s003.docx]
